# Ethnic differences on long term outcomes of polypoidal choroidal vasculopathy after predominantly bevacizumab monotherapy

**DOI:** 10.1186/s12886-022-02551-3

**Published:** 2022-07-28

**Authors:** Aaron Yap, Nancy Wang, David Squirrell

**Affiliations:** 1 Department of Ophthalmology, The University of Auckland, 85 Park Road, Grafton, Auckland, 1051 New Zealand; 2 Department of Ophthalmology, Auckland District Health Board, Auckland, New Zealand

**Keywords:** Polypoidal choroidal vasculopathy, Bevacizumab, Treatment outcomes, Real-world study, Long term

## Abstract

**Background:**

A 3-year single-centre, retrospective, comparative, non-randomized cohort study to describe the long-term outcomes of treatment-naïve, Caucasian and non-Caucasian eyes with polypoidal choroidal vasculopathy (PCV) after treatment with predominantly Bevacizumab monotherapy or in combination with rescue photodynamic therapy (PDT).

**Methods:**

Demographics, visual outcomes, optical coherence tomography (OCT) and treatment data were collected up to 3 years after the first visit. Stratified analysis according to ethnicity and baseline vision was performed to identify factors predictive of long-term visual improvement and maintenance.

**Results:**

A total of 89 eyes with PCV were identified, of which 14 received rescue verteporfin PDT. There was an equal distribution between Caucasian and non-Caucasian individuals. Non-Caucasians present at a younger age (67.3 vs. 76.0 years, *p* = 0.002), have a higher proportion of foveal involvement (80.9%, vs.54.2% *p* = 0.007), choroidal hyperpermeability (50% vs 25.8%, *p* = 0.013) and lower baseline visual acuity (53.1 vs. 63.3 letters, *p* = 0.008). Mean visual acuity (VA) gain was + 8.9 letters and + 5.0 letters at 1 and 3 years of follow-up, respectively. Non-Caucasian individuals had a lower mean final visual acuity (VA) (54.7 vs. 70.5, respectively; *P* < 0.001) and net gain in VA (+ 2.0 vs. + 7.6 letters, *p* = 0.581) compared to Caucasian individuals. The mean total number of injections given over 3 years was 14.

**Conclusions:**

Most patients treated with predominantly Bevacizumab anti-vascular endothelial growth factor (VEGF) monotherapy achieved sustained visual acuity gains out to 3 years. Due to ethnic-specific differences in presenting PCV phenotypes, non-Caucasians presented with lower baseline VA and had poorer long-term visual outcomes.

**Supplementary Information:**

The online version contains supplementary material available at 10.1186/s12886-022-02551-3.

## Background

Since its first description in the 1990s [1], the literature on Polypoidal Choroidal Vasculopathy (PCV) has expanded rapidly. Attempts to categorise PCV according to features observed on multimodal imaging across different contexts have yielded classifications that are increasingly complex and not readily translated to clinical practice. As a result, PCV is best considered as a wide spectrum of disease, within which there are a number of well recognised and characteristic diagnostic criteria [2]. Whilst the pivotal trials, EVEREST and PLANET, have provided important guidance with respect to the management of PCV, they were predominantly conducted in large Asian centres, raising the possibility that the results may have limited generalisability to mixed populations with wider phenotypic variations of PCV [3, 4].

It has recently been recognised that there may be distinct differences between the phenotypes of PCV seen in Caucasian and non-Caucasian eyes [5]. However, little is known about whether this translates into differences in long term visual outcomes and response to available treatments. Auckland, with its diverse ethnic population, facilitates comparison of treatment outcomes across patients from different ethnic groups treated at a single tertiary centre [6]. The aim of this study was to evaluate the three-year visual outcomes of patients with PCV treated with predominantly Bevacizumab monotherapy and to identify if ethnic-specific differences in the presenting PCV phenotypes influenced long-term visual outcome.

## Methods

**Fig. 1:**
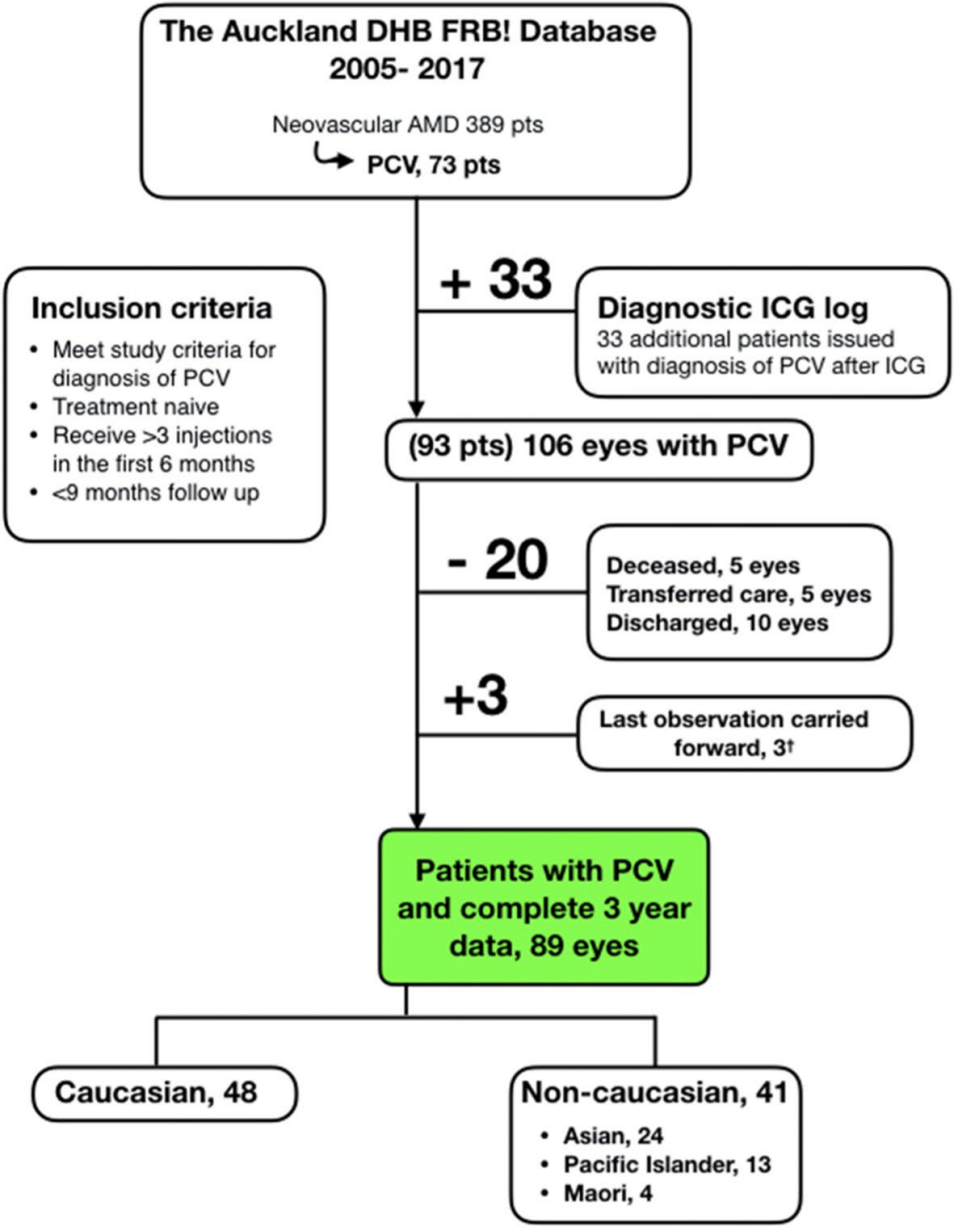
Flowchart Outlining the Patient Selection Process and Reasons for Patient Exclusion and Lost to Follow Up. FRB!: Fight Retinal Blindness, RPE: Retinal Pigment Epithelium, PCV: Polypoidal choroidal vasculopathy, ICG: Indocyanine green angiography, OCT: Optical coherence tomography. † last observation carried forward for three patients that were discharged due to poor visual prognosis

We performed an observational cohort study of the outcomes of eyes diagnosed with PCV, within the Auckland District Health Board, New Zealand (NZ) Fight Retinal Blindness (FRB!) database. The patient selection process is detailed in Fig. 1. Not all clinicians utilise FRB!, so the indocyanine green angiography (ICGA) diagnostic logs of the eye department were also reviewed to ensure that all patients diagnosed in the unit with PCV between 2005 and 2017 were included. All patients had to be treatment naïve and meet predefined ICGA or OCT diagnostic criteria for PCV [3, 7]. Both peripapillary and macular polypoidal lesions were included in this study. Only eyes with complete 3 year follow up data were included in the final analysis. Reasons for non-completion and baseline demographics of non-completers were recorded. The cohort was divided into two groups, Caucasian and non-Caucasian, to investigate the influence that ethnicity may play in both the presentation and response to treatment.

**Fig. 2:**
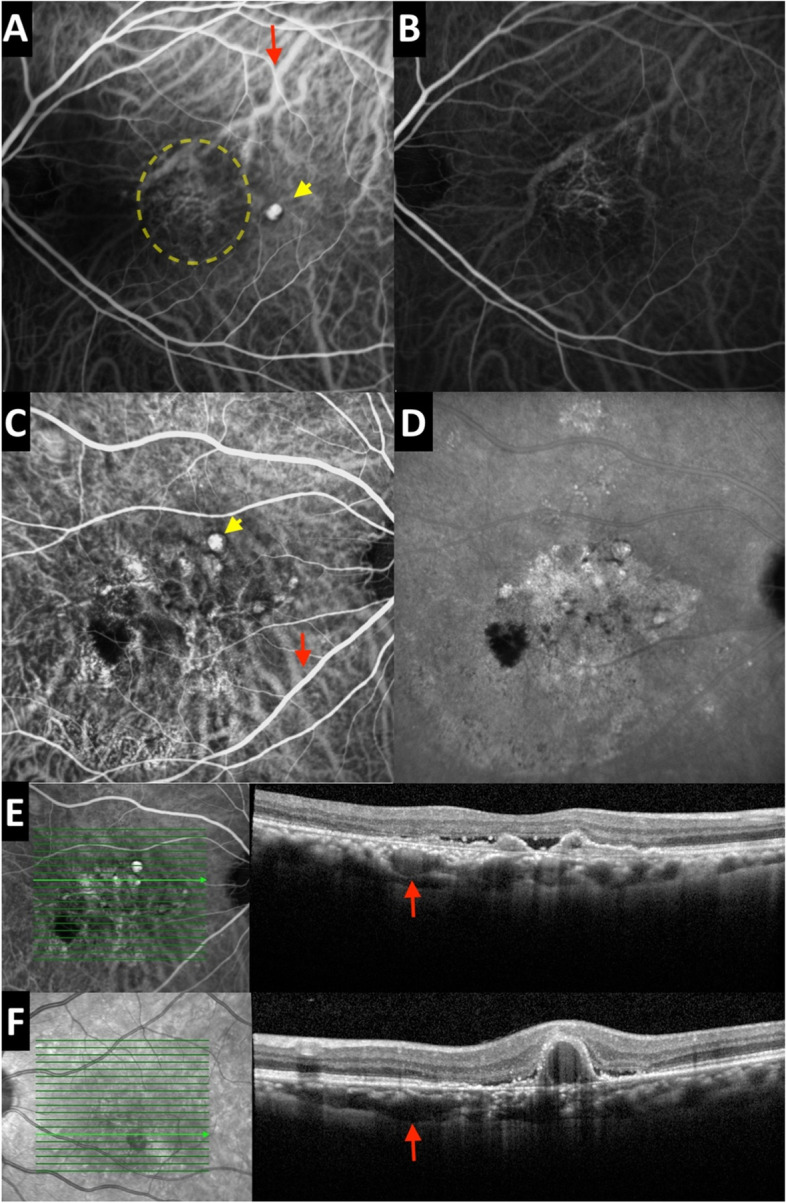
Red arrows highlight pachyvessels on ICGA and OCT. Polyps are indicated by yellow arrows. **A**, **B** ICGA frames captured at 1 minute of the same patient, taken 11 months apart showing a small branching vascular network and regression of the polyp. **C**, **D**, **E** are taken from the same patient. **C** Branching vascular network with string of polyps. **D** Late phase ICG demonstrating multifocal areas of hyperfluoresence with blurred margins, indicative of choroidal vascular hyperpermeability. **E** OCT scan showing thumb-like PED, subretinal fluid and underlying pachyvessels. **F** OCT scan showing a sub-RPE ring like lesion

**Fig. 3:**
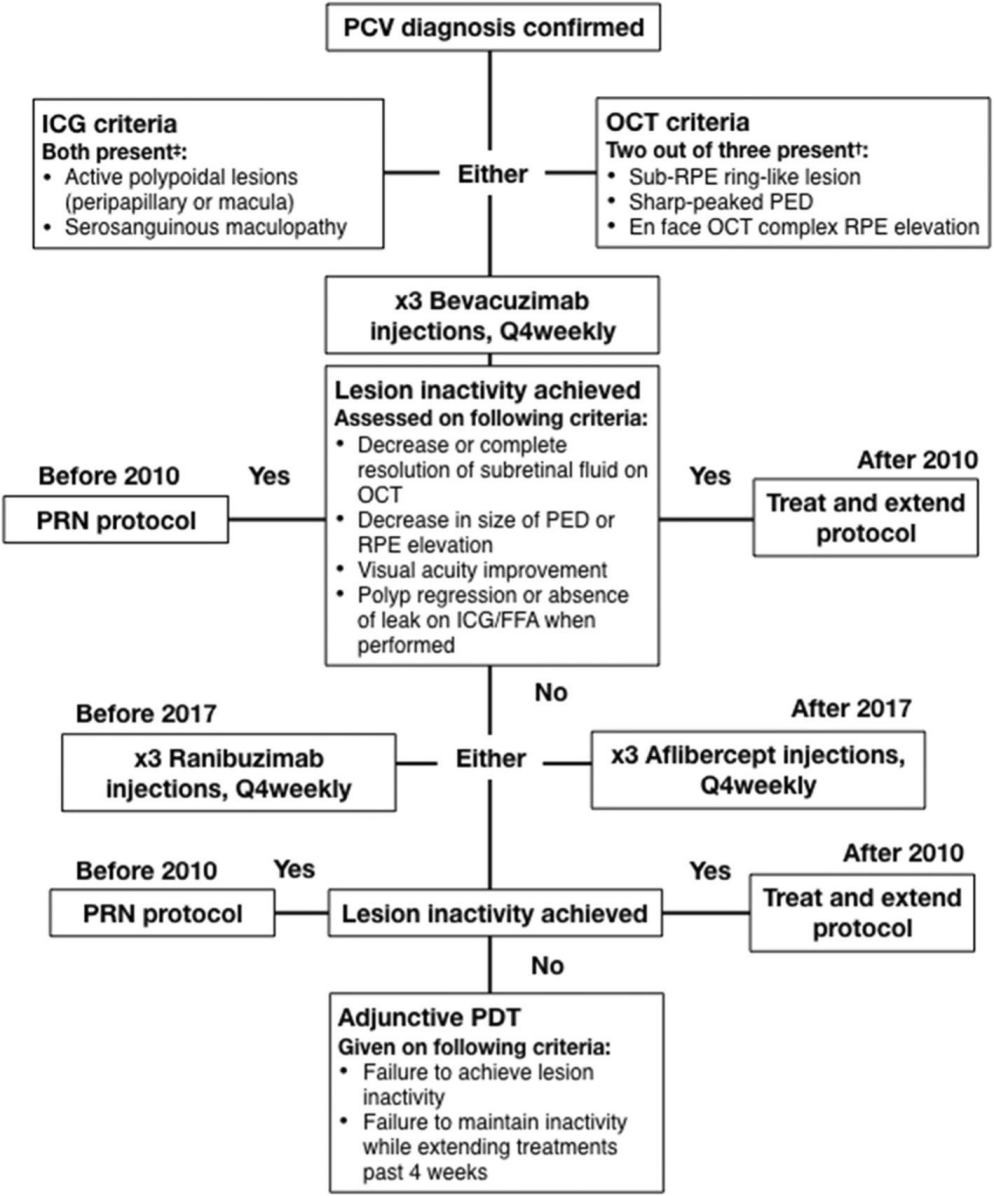
Flowchart Outlining the Diagnostic Criteria for Polypoidal Choroidal Vasculopathy and Treatment Algorithms. RPE: Retinal Pigment Epithelium, PCV: Polypoidal choroidal vasculopathy, ICG: Indocyanine green angiography, OCT: Optical coherence tomography. † per Non-ICG Diagnostic Criteria from the Asia-Pacific Ocular Imaging Society PCV Workgroup [7]. ‡ as per ICG diagnostic criteria in EVEREST [3]

Briefly, the FRB! database is a cloud-based, record-keeping software that stores data from each clinical visit [8]. Entry datapoints include best- corrected VA, lesion subtype (classic, occult, PCV), greatest linear diameter, lesion activity, treatment, and any ocular adverse events. Visual acuity is recorded in equivalent logarithm of the minimum angle of resolution (logMar) letters. Although management decisions, including frequency of visits and treatment method, was determined by the treating clinician, the overarching treatment algorithm is outlined in Fig. 3. Lesion activity status was graded by the treating physician based on OCT, ICGA, or a combination of both, at each visit. Choroidal vascular hyperpermeability was defined as multifocal areas of hyperfluoresence with blurred margins in the late phase of ICG (Fig. 2) [9]. Pachychoroid phenotype was defined as the presence of either 1) Subfoveal choroidal thickness greater than 270 um, or 2) Pachyvessels [10, 11]. The OCT description of pachyvessels is dilated outer choroidal vessels causing attenuation of the choriocapillaris and Sattler vessels (Fig .2) [12]. Pachyvessels on ICGA would appear as dilated choroidal vessels that extended along the entire course of the vessel back to at least one of the ampullae of the vortex vein (Fig. 2) [13].

Baseline scans were retrospectively analysed by the authors for the purpose of recording the baseline characteristics of PCV lesions and to ensure that the diagnosis of PCV met the predefined diagnostic criteria (Fig. 3). In cases of missed or incomplete entries, data was corroborated from the physical records.

### Outcome measurements

The primary study outcome was the mean VA over 3 years after initiating treatment. Secondary outcomes were the proportion of eyes that achieved lesion inactivity, time to inactivity, median number of injections per year, and proportion receiving photodynamic therapy (PDT), Aflibercept or Ranibizumab. Self-reported ethnicity data was extracted from the clinical records. Individuals who identified with more than one ethnicity were routinely classified to a single ethnicity based on a defined prioritized order [14]. Stratified analysis according to Caucasian and non-Caucasian ethnicity, and baseline vision were performed. Patients that were discharged following poor visual prognosis had their last visual acuity measured brought forward for final analysis.

### Statistical analysis

Descriptive data are presented as mean (standard deviation), mean (95% confidence interval), or number (percentage). The Student T-test, Fisher’s test, Wilcoxon test was used to compare the differences in baseline characteristics and final visual outcomes between different groups. *P*-value of less than < 0.05 was considered statistically significant. All statistical analysis was performed using IBM SPSS© Statistics (Release 27.0.1.0).

Kaplan Meier survival curves were used to display the time to inactivity. Locally Weighted Scatterplot Smoothing curves (LOESS) were used to plot the visual acuity trends for different ethnic groups and eyes with varying baseline vision. All graphs were produced using GraphPad Prism© Version 9.2.0 (283).

## Results

### Study participants

From the Auckland DHB FRB! database and ICGA diagnostic logs, a total of 89 eyes met the eligibility criteria specified in Fig. 3. Baseline characteristics of the entire cohort, including those that were later excluded, are outlined in Table 1. Fifty-three percent of patients were NZ European, 27% Asian, 13% Pacific Islander and 5% were NZ Māori. To examine for differences in ethnicity, patients were categorised into Caucasian (53%) and non-Caucasian eyes (47%). The mean baseline VA was higher in Caucasian participants compared with non-Caucasian participants (63.3 ± [SD 15.5 letters] vs. 53.1 ± [SD 21.5 letters], respectively; *P* = 0.02). Non-Caucasian individuals were more likely to present at a younger age with larger lesions which were associated with branching vascular networks. Their lesions also had a predilection to involve the fovea. Baseline ICGA, where available, revealed that non-Caucasian individuals were also more likely to have strings or clusters of polyps, which filled early in the ICGA transit. The rate of choroidal vascular hyperpermeability (CVH) was higher in the non-Caucasian group.

**Table 1:** Baseline characteristics of all patients diagnosed with PCV including those that were later excluded due to lost to follow up

	Caucasian	Non-Caucasian	*p*-value	Total cohort
No. of eyes (%)	59 (55.6%)	47 (44.3%)	–	106
No. bilateral (%)	9 (15.3%)	4 (8.5%)	–	13 (12.3%)
Mean age (SD), years	76.0 (9.7)	67.3 (9.1)	0.002^d^	72.1 (10.3)
Gender, male (%)	19 (32.2%)	23 (48.9%)	0.11^c^	42 (39.6%)
Mean baseline VA (SD), letters	63.3 (15.5)	53.1 (21.5)	0.008^e^	58.8 (19.0)
VA ≥70 letters (20/40 Snellen)	23 (38.9%)	11 (23.4%)	0.098^c^	34 (32.1%)
VA ≤ 35 letters (20/200 Snellen equivalent)	4 (6.8%)	9 (19.1%)	0.074^c^	13 (12.3%)
Presence of CVH, no (%)	8 (25.8%)	17 (50.0%)	0.013^c^	25 (34.2%)
Pachychoroid phenotype, no (%)	29 (60.4%)	30 (73.2%)	0.262^c^	59 (66.3%)
Presence of BVN, no (%)	25 (42.4%)	31 (66.0%)	0.019^c^	56 (52.8%)
Foveal involvement, no. (%)	32 (54.2%)	38 (80.9%)	0.007^c^	70 (66.0%)
Cluster/String Configuration, no. (%)	29 (49.2%)	32 (68.1%)	0.075^c^	61 (57.5%)
Solitary polyp Configuration, no. (%)	16 (27.1%)	7 (14.9%)	0.158^c^	23 (21.7%)
Early filling^a^, no. (%)	8 (13.6%)	18 (38.3%)	0.006^c^	26 (24.5%)
Late filling^b^, no. (%)	30 (50.8%)	18 (38.3%)	0.240^c^	48 (45.3%)
Mean (SD) Central Retinal Thickness, microns	384 (160)	423 (251)	0.839^e^	400 (204)
Mean GLD (SD), microns	2750 (1600)	4018 (1904)	0.013^e^	3452 (1870)

*SD* Standard deviation, *GLD* Greatest linear diameter, *IQR* Interquartile range, *VA* Visual acuity, *BVN* Branching vascular network, *CVH* Choroidal vascular hyperpermeability

^a^ Polyp fill during arteriolar phase of ICG

^b^ Polyp fill during late venous phase of ICG

^c^ Fisher exact test

^d^ Student t-te**s**t

^e^ Wilcoxon rank-sum test

### Non-completion rate

Seventeen patients were excluded. The reasons included: Patient death, the patient elected to seek care outside of the public hospital, the patient was referred from to Auckland from another unit solely for an ICGA, the patient left Auckland during treatment (Fig. 1). The demographics of this population is outlined in Table 2. This represents 17% of all eyes that were commenced on treatment for PCV in our unit. The mean duration of follow up of the non-completers was 20.5 months and the final mean VA was 71.4 letters. Of those that were discharged, three patients had their last measured VA brought forward after discharge with poor visual prognosis (VA ≤ 35 letters). The remaining four patients were discharged after a long period of inactivity. These patients, along with those deceased, all had a last measured VA of greater than 60 letters. Given the unpredictability of their future visual outcome, their last measured VA was not carried forward for final analysis. The baseline demographics of those eyes that were excluded were broadly comparable to the study cohort (Table 2 vs Table 1), including mean baseline vision (61.5 ± SD 13.3 vs. 58.8 ± SD 19.0, *p* = 0.641).

**Table 2:** Demographics of patients that were lost to follow up and excluded due to incomplete data

Ethnicity no. (%)	
*Caucasian*	12 (71%)
*Non-Caucasian*	5 (29%)
Gender no. (%)	
*Male*	6 (35%)
*Female*	11 (65%)
Mean age (SD), years	73.1 (12.5)
Mean baseline VA (SD), letters	61.5 (13.3)
Mean final VA at last visit (SD), letters	71.4 (9.4)
Mean Follow Up Time (SD), months	20.5 (5.7)

*SD* Standard deviation, *VA* Visual acuity

### Visual outcomes

**Fig. 4:**
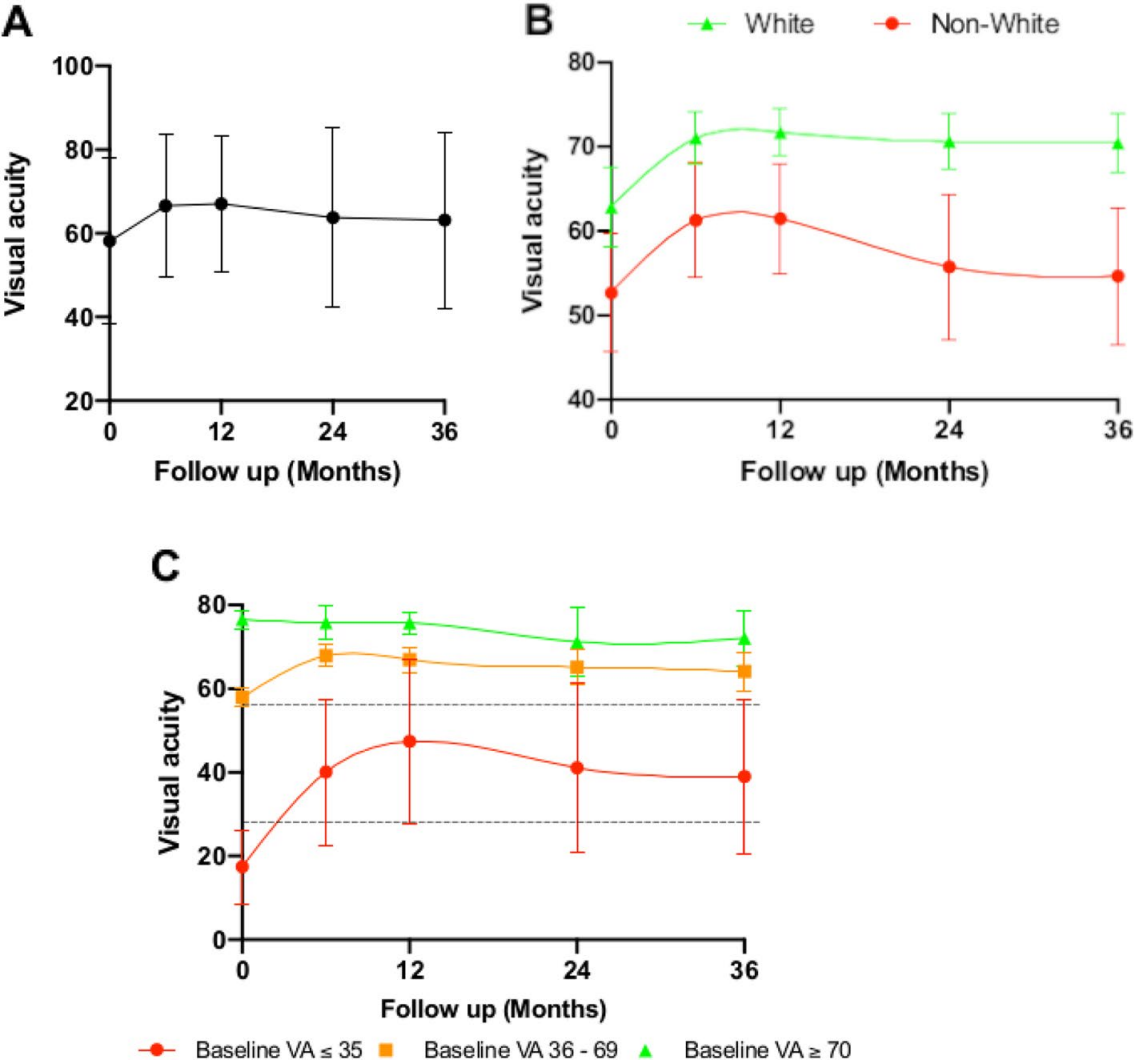
**A** Mean ± standard deviation, best corrected visual acuity in LogMar Letters over the 3 years of follow up. **B** LOESS regression curve of mean visual acuity (VA) ± 95% confidence interval, stratified according to ethnicity. **C** LOESS regression curve of mean visual acuity (VA) ± 95% confidence interval, stratified according to baseline VA, the dotted lines represent the cut-off values of 70 and 35 letters

The mean visual acuity for all eyes for each year is displayed in Fig. 4A. Overall, there was a 8.9 letter gain from baseline at 12 months. At 3 years, there was a mean gain of 5.0 letters compared to baseline (63.2 ± [SD 21.0] letters vs 58.2 ± [SD 19.9 letters], respectively, *p* = 0.021). All other visual outcomes are outlined in Table 3. The only significant difference between ethnicities was that non-Caucasian individuals (54.7 ± SD 25.8 letters) had a lower mean final VA compared to Caucasian individuals (70.5 ± [SD 25.0 letters], *p* < 0.001). Figure 4B plots the mean visual change over 3 years for Caucasian and non-Caucasian individuals. The largest gains in vision occurred within the first year for both Caucasian and non-Caucasian individuals, with both groups achieving a mean improvement in visual acuity gain of 8.8 letters.

**Table 3:** Comparison of visual outcomes between different ethnic groups

	12 months	36 months	36 months (Caucasian)	36 months (Non-Caucasian)	*p*-value
No. of eyes	89	89	48	41	–
Mean ± SD final VA, letters	67.0 ± 16.4	63.2 ± 21.0	70.5 ± 12.0	54.7 ± 25.8	< 0.001^b^
Mean ± SD CRT, microns	307.3 ± 146.5	295.4 ± 122.2	269.9 ± 77.0	327.0 ± 157.2	0.269^b^
Mean change in VA letters (95% CI)	+ 8.9 (2.7 to 10.5)	+ 5.0 (− 2.7 to 8.8)	+ 7.6 (3.2 to 12.0)	+ 2.0 (− 5.8 to 9.8)	0.581^b^
Proportion with ≥15-letter gain	24 (27.0%)	29 (32.6%)	17 (35.4%)	12 (29.3%)	0.651^c^
Proportion with ≤15-letter loss	84 (94.4%)	78 (87.6%)	44 (91.7%)	34 (82.9%)	0.333^c^
VA ≥70 letters (20/40 Snellen equivalent)	48 (53.9%)	47 (52.8%)	33 (68.8%)	14 (34.1%)	0.001^c^
Mean total ± SD, no. of injections	5.8 ± 2.9	13.8 ± 8.1	14.0 ± 8.1	13.6 ± 8.1	0.833^d^
Received PDT, no. (%)	5 (5.6%)	14 (15.7%)	4 (8.3%)	10 (24.4%)	0.08^c^
Mean time to PDT (SD), months	–	–	30 (15.2)	19.1 (15.6)	0.185^d^
Polyp regression, no (%)^a^	2/24 (8.3%)-	12/24 (50.0%)	7/10 (70.0%)	5/14 (35.7%)	0.214^c^
Mean time to Polyp Regression (SD), months^a^	–	–	45.3 (33.5)	34 (19.9)	0.519^d^
Received Aflibercept or Ranibizumab, no. (%)	6 (6.7%)	15 (16.9%)	11 (22.9%)	4 (9.8%)	0.155^c^

*CRT* Central Retinal Thickness, *VA* Visual acuity, *CI* Confidence interval, *SD* Standard deviation, *PDT* Photodynamic therapy

^a^Findings based on 24 patients that underwent repeat ICGA

^b^Wilcoxon Rank-Sum test

^c^Fisher’s exact test

^d^Student t-test

The mean visual change over 3 years, stratified according to baseline visual acuity, is shown in Fig. 4C. Eyes were stratified into 3 groups according to their presenting baseline VA: (1) good baseline vision of 70 letters or more (27 eyes), (2) moderate baseline vision of between 36 and 69 letters (50 eyes), and (3) low baseline vision of 35 letters or fewer (12 eyes). The mean VA of eyes with good baseline vision was initially 74.3 letters (SD 7.2), dropping down to 70.2 (SD 17.4) letters at year three. The presenting mean VA and final VA of eyes with intermediate baseline VA was similar to that of the entire cohort, increasing from 58.0 letters (SD 12.3) at baseline to 65.6 letters (SD 18.5) at 3 years; an increase of 7.6 letters. Eyes with poor baseline vision had a mean baseline VA of 22.4 letters (~ 20/400) and experienced an increase of 23.1 letters (~ 20/160) at 3 years; all improvement occurred in the first year of treatment. Overall, those who presented with good vision had a better final VA at 3 years compared to those who started off with poor vision. (70.2 ± [SD 17.4] vs. 45.5 ± [SD 28.8], *p* = 0.007).

### Secondary outcomes

**Fig. 5:**
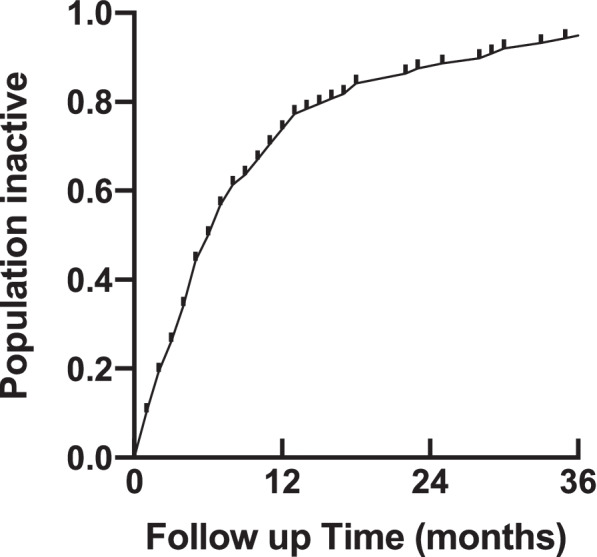
Kaplan Meier Survival Curve showing the proportion of patients achieving lesion inactivity

Kaplan-Meier survival curve representing the proportion of eyes achieving inactivity is shown in Fig. 5. Seventy-four percent of patients achieved lesion inactivity by 12 months, rising to 88% at 24 months. Overall, the mean central retinal thickness dropped by 82 μm within the first year (386 ± [SD 176 μm] vs 307 ± [SD 147 μm], paired T-test *p* < 0.001) and remained stable until year three (295 ± [SD 122 μm]) (Table 3).

The median number of injections delivered in the first year was 6 and ranged between 3 to 4 injections in the second and third year. The total mean number of injections given to study completers at the end of 3-years was 13.8. There was a higher mean total number of injections given to those that had a final vision of greater than 70 letters at 3 years, compared to those with vision worse than 35 letters (13.2 ± 7.9 vs 9.6 ± 5.9, *p* = 0.182, respectively).

Seventy-three (82%) patients underwent baseline ICGA and 6 (6.7%) patients underwent OCT enhanced depth imaging (EDI). Of the 24 patients that had follow up ICGA, a higher proportion in the Caucasian group achieved polyp regression. A higher proportion of non-Caucasian eyes received PDT (24% vs 8%), but this finding was not statistically significant. The mean time to application of PDT was 30 months for Caucasians and 19 months for non-Caucasians patients.

## Discussion

This study evaluates the real-world outcomes of patients with PCV in a single tertiary unit that serves an ethnically diverse population predominantly with Bevacizumab monotherapy. The largest gain in visual acuity occurred in the first 12 months with mean increase of 8.8 letters. At 36 months, Caucasians had sustained superior visual gains (+ 7.6 letters) compared to non-Caucasians patients (+ 2.0 letters). Non-Caucasians presented with worse visual acuity and experienced poorer final visual outcomes at 36 months follow up, and this could be attributable to the differences in their presenting PCV phenotype as demonstrated on multimodal imaging.

All patients in this study were managed according to treatment algorithm outlined in Fig. 2. The treat and extend approach was used as it has been demonstrated to produce significant visual improvement, whilst minimising the burden of treatment upon the patient and healthcare system [15–17]. Whilst all clinicians will have broadly followed this algorithm, in the real world setting the exact interpretation of the treatment plan is ultimately determined by the physician after taking into consideration multiple patient, societal and resource dependant factors. Thus, in a study such as this there is an inevitable degree of treatment heterogenicity. As a result, comparison between groups can be problematic and further studies are required to validate the findings of the current study. A further complicating factor was that Aflibercept became available during this study, but its use was limited to those patients that failed to respond to Bevacizumab. As such, it became the treatment of choice for treating patients with persistent activity from PCV lesions [18]. However, this treatment was not funded for those patients with visual acuity below 6/36 (45 logMar Letters), a limitation which affected more patients in the non-Caucasian cohort compared to the Caucasian cohort. This likely explains the difference in the volumes and types of anti-VEGF used in the two groups. Finally, referrals for photodynamic therapy were all outsourced to private providers, and the lengthy approval process to secure this treatment likely contributed to the long delays for this treatment to be administered.

The visual outcomes in the current study were compared with previously published real-world observational studies for PCV as illustrated in Table A1. Whilst the data is very heterogenous, three key themes emerge; 1. All long-term studies demonstrate peak visual gains at 1 year, with a subsequent decline in the following maintenance years, regardless of therapeutic regimen [19–23]. 2. There is a paucity of long-term data from Caucasian populations with PCV and thus there are few comparisons of visual outcomes based on ethnicity. 3 Combination therapy with PDT, at least in non-Caucasians, is associated with superior visual outcomes despite fewer injections [23–26].

The total median number of six injections in the first year lies upon the upper range of studies based in Asia but is consistent with Singaporean and Australian studies (Table A1). Under treating in terms of lower frequency of anti-VEGF injection, has been shown to be a strong determinant of poor visual outcomes in long term studies of patients with neovascular AMD [24, 27, 28]. The mean number of injections administered over 36 months is similar to that of the anti-VEGF monotherapy arm in the EVEREST study over 24 months, suggesting an element of under treatment in our study cohort. It has been well described that anti-VEGF use in real world practice often differs from the strict regimen used in RCTs, owing to factors such as resource constraints and heterogenous treatment algorithms [29, 30].

Head-to-head comparison with landmark trials, such as EVEREST and PLANET, is confounded by heterogenicity of treatment algorithms among individual trials and the real world setting. Whilst PLANET found Aflibercept monotherapy was non-inferior to Aflibercept combined with PDT rescue, EVEREST II demonstrated superior visual gains with combination therapy compared with Ranibizumab monotherapy [3, 4]. Despite these differences, a higher proportion of patients in the monotherapy arm of PLANET achieved disease inactivity after three loading injections compared to EVEREST, suggesting that Aflibercept may be more effective in treating individuals with PCV than Ranibizumab [3, 4]. These findings may not be generalisable towards other treatment centres that employ Bevacizumab as first-line therapy. Despite this, Bevacizumab remains the first-line agent to treat neovascular AMD and PCV in many health economies due to its cost-effectiveness. This study provides reassurance that Bevacizumab monotherapy, with rescue PDT or Aflibercept reserved for non-responders, yielded good visual outcomes in the long-term.

Consistent with other published data, we found that both the demographics and the morphological characteristics of PCV differed according to ethnicity. It is well described that the prevalence of PCV among those diagnosed with neovascular AMD is higher in Asians (20–60%) [31–33] compared to Caucasians (4–10%) [34–37]. PCV also tends to present a decade later in Caucasians [34, 36–38] compared to Asians [32, 39–44] and whilst there is a male predominance in Asians [32, 40–43], the opposite is seen in Caucasians [36, 38, 44, 45]. In addition to the demographic differences, there also appears to be distinct anatomical differences between ethnic groups. Whilst Asians tend to present with large lesions and BVNs that have a predilection to involve the central macula [32, 41, 42, 46], Caucasians tend to present with smaller lesions which often spare the fovea [35–38, 44, 45, 47]. Our findings mirror those previously reported; Caucasians tended to be older, presenting with solitary macular polyps or clusters which filled in the venous phase, with no accompanying BVN, while non-Caucasians were more likely to have strings or clusters in the central macula area with large associated BVNs. The finding of choroidal hyperpermeability was also more prevalent in the Non-Caucasian group [5]. In the current study, Caucasian patients tend to manifest a different disease phenotype, presented with better visual acuity and reported superior long-term visual outcomes compared to Non-Caucasian patients. These findings then raise the possibility that the observed differences in clinical presentation of PCV may influence how the two ethnic groups respond to treatment.

This study has limitations inherent to observational studies that should be acknowledged. The recruitment of patients using ICGA logs and a separate analysis of excluded patients were purposefully done to mitigate selection bias. Unlike randomised controlled trials, case and treatment selection were performed without reference to an adjudication centre or strict protocols. Whilst the cost of treatment is fully covered by the public health system in New Zealand, other barriers towards healthcare access such as health literacy and socioeconomic status that potentially affect treatment frequency and modality were not explored in this study [48, 49]. The data presented therefore has a lower internal validity but is still meaningful because they are an accurate representation of decisions made in real-world practice.

The lack of standardised investigation protocol may have led to an underestimation of the multimodal imaging characteristics of PCV. Indocyanine green angiography is not routinely preformed on all individuals who present with neovascular AMD, nor is it routinely used to monitor for polyp regression after treatment. Instead, it is reserved for cases with a high suspicion of PCV and to help determine if the patient would benefit from PDT or focal argon laser therapy. This practice is likely to have led to the unjustified exclusion of some patients with PCV being managed in our unit and an underestimation of branching vascular networks, polyp clusters and rates of poly regression. Enhanced depth imaging OCT is also not routinely requested; hence, most patients were classified as pachychoroid based on the presence of pachyvessels.

In the presence of all three OCT-based criteria outlined in Fig. 2, the area under the receiver operating characteristic curve (AUC) is 0.90 [7]. In the presence of two of three criteria, the AUC drops to 0.82–0.89. As the departmental imaging protocol does not include en-face OCT scans that screen for complex RPE elevations, the inclusion threshold was lowered to two criteria. Although this allows for a small proportion of false positive cases, it is another reflection of real-world practice where access to certain imaging modalities is limited.

In conclusion, Caucasian patients have a preponderance to present with solitary, peripapillary polypoid lesions and have good visual outcomes up to 3 years with predominately Bevacizumab anti-VEGF monotherapy. Conversely, Non-Caucasian patients, who are more likely to present with PCV at a younger age with poor visual acuity and foveal involvement, had poorer visual outcomes inviting discussion as to whether earlier PDT or alternative anti-VEGF agents should be offered to this group.

## Supplementary Information

**Additional file 1: Table A1.** Comparison of Clinical Outcomes in the Current Study with Previously Published Real-World Observational Studies for Polypoidal Choroidal Vasculopathy Composing Different Ethnic Compositions Treated with Anti-Vascular Endothelial Growth Factor Monotherapy or Combination Therapy with Verteporfin Photodynamic Therapy.

## Data Availability

The datasets generated and analysed during the current study are not publicly available due to potential compromise to individual privacy but are available from the corresponding author on reasonable request.

## Abbreviations

AMD: Age related macular degeneration
Anti-VEGF: Anti vascular endothelial growth factor
BVN: Branching vascular network
CI: Confidence interval
CNVM: Choroidal neovascular membrane
CRT: Central retinal thickness
EDI: Extended depth imaging
FRB!: Fight Retinal Blindness
ICG: Indocyanine green angiography
IQR: Interquartile range
OCT: Optical coherence tomography
PCV: Polypoidal choroidal vasculopathy
PDT: Photodynamic therapy
PED: Pigment Epithelial Detachment
RPE: Retinal Pigment Epithelium
RWS: Real World Studies
SD: Standard deviation
VA: Visual acuity
