# Supplementary material for: Ethnic differences on long term outcomes of polypoidal choroidal vasculopathy after predominantly bevacizumab monotherapy
Source: BMC Ophthalmol. 2022 Jul 28;22:325. doi: 10.1186/s12886-022-02551-3 (PMC9330682; doi:10.1186/s12886-022-02551-3)
Supplement: Supplementary file 1 — Additional file 1: Table A1. Comparison of Clinical Outcomes in the Current Study with Previously Published Real-World Observational Studies for Polypoidal Choroidal Vasculopathy Composing Different Ethnic Compositions Treated with Anti-Vascular Endothelial Growth Factor Monotherapy or Combination Therapy with Verteporfin Photodynamic Therapy. [file 12886_2022_2551_MOESM1_ESM.docx]

Table A1. Comparison of Clinical Outcomes in the Current Study with Previously Published Real-World Observational Studies for Polypoidal Choroidal Vasculopathy Composing Different Ethnic Compositions Treated with Anti-Vascular Endothelial Growth Factor Monotherapy or Combination Therapy with Verteporfin Photodynamic Therapy.

| Author, number of eyes | Ethnicity of Population | Baseline VA, logMar letters | VA 1 year, logMar letters | VA 3 years, logMar letters | Mean total number of injections, follow up duration, anti-VEGF agent | Photodynamic therapy |
| --- | --- | --- | --- | --- | --- | --- |
| Teo, 193^1^ | Caucasian 65  Non-Caucasian 115  Undisclosed 13 | 46^†^ - 60^‡^ | 67.5^†^ - 70^‡^ | - | 4.3^‡^ – 6.4^†^ over 12 months  Predominantly Bevacuzimab | Mean of 1.23 treatments within initial 3 months |
| Current cohort, 48 | Caucasian | 62.9 | 71.7 | 70.5 | 14 over 3 years | 4 had PDT, none in the first year |
| Chehab, 50^2^ | Caucasian | 59.9^¶^ | 60.4^¶^ | - | 8.4-8.6 over 24 months  Ranibizumab/Aflibercept | 15 had PDT, with 6 in the first 6 months. |
| Gharehbagh, 29^3^ | Caucasian | 62^¶^ | 66^¶§^ | - | 7.6 over 22 months  Aflibercept | 7 had PDT |
| Current cohort, 41 | Non-Caucasian | 52.7 | 61.5 | 54.7 | 13.6 over 3 years | 10 had PDT, 5 in the first year |
| Fenner, 199^4^ | Non-Caucasian | 41.6^†^–45.1^‡^ | 48.2^†^–55.9^‡^ | - | 5.0^†^-5.6^‡^ over 12 months  Predominantly Bevacuzimab | 100 had PDT, 66 in the first 3 months |
| Miyata, 61^5^ | Non-Caucasian | ~65  (0.4^†^-0.41^‡^) | ~70-73  (0.26^‡^-0.29^†^) | ~68  (0.32^†^-0.33^‡^) | 5.3^‡^ – 9.2^†^ over 5 years  Ranibizumab | 20 had initial PDT |
| Wataru, 53^6^ | Non-Caucasian | ~57 (0.55) | ~70^§^ | ~70^§^ | 7.51 over 5 years  Ranibizumab/Aflibercept | All had initial PDT |
| Chang, 31^7^ | Non-Caucasian | ~60 (0.52) | ~63 (0.46) | ~47 (0.76) | 8.8 over 53 months  Ranibizumab/Bevacuzimab | - |
| Kang, 42^8^ | Non-Caucasian | ~45 (0.78) | ~53^§^ | ~53^§^ | 6.42 over 5 years  Ranibizumab/Bevacuzimab | All had initial PDT |
| Hikichi, 66^9^ | Non-Caucasian | ~67 (0.34) | ~75^§^ | ~70 (0.32) | 21.5 over 6 years  Ranibizumab | - |

~ Converted from logMar decimal to logMar letters

† Combination anti-VEGF and PDT therapy group

‡ Anti-VEGF monotherapy group

§ Inferred from plotted points on graph

¶ Early Treatment Diabetic Retinopathy Study (ETDRS) letters

1. Chong Teo KY, Squirrell DM, Nguyen V, Banerjee G, Cohn A, Barthelmes D, et al. A Multicountry Comparison of Real-World Management and Outcomes of Polypoidal Choroidal Vasculopathy: Fight Retinal Blindness! Cohort. Ophthalmol Retina. 2019;3(3):220-9.

2. El Chehab H, Kodjikian L, Lagenaite-Desmaizère C, Agard E, De Bats F, Mathis T, et al. Idiopathic polypoidal choroidal vasculopathy in Caucasians: The POLYON real-life study in 50 naive patients. Eur J Ophthalmol. 2020;30(5):948-55.

3. Gharehbagh SS, Subhi Y, Sørensen TL. Efficacy of aflibercept for polypoidal choroidal vasculopathy in Caucasians. Acta Ophthalmol. 2018;96(1):e94-e5.

4. Fenner BJ, Ting DSW, Tan ACS, Teo K, Chan CM, Mathur R, et al. Real-World Treatment Outcomes of Age-Related Macular Degeneration and Polypoidal Choroidal Vasculopathy in Asians. Ophthalmol Retina. 2020;4(4):403-14.

5. Miyata M, Ooto S, Yamashiro K, Tamura H, Hata M, Ueda-Arakawa N, et al. Five-year visual outcomes after anti-VEGF therapy with or without photodynamic therapy for polypoidal choroidal vasculopathy. British Journal of Ophthalmology. 2019;103(5):617-22.

6. Wataru K, Sugiyama A, Yoneyama S, Matsubara M, Fukuda Y, Parikh R, et al. Five-year outcomes of photodynamic therapy combined with intravitreal injection of ranibizumab or aflibercept for polypoidal choroidal vasculopathy. PLOS ONE. 2020;15(2):e0229231.

7. Chang YS, Kim JH, Kim KM, Kim JW, Lee TG, Kim CG, et al. Long-term outcomes of anti-vascular endothelial growth factor therapy for polypoidal choroidal vasculopathy. Journal of Ocular Pharmacology and Therapeutics. 2016;32(4):219-24.

8. Kang HM, Kim YM, Koh HJ. Five-year follow-up results of photodynamic therapy for polypoidal choroidal vasculopathy. American journal of ophthalmology. 2013;155(3):438-47. e1.

9. Hikichi T. Six-year outcomes of antivascular endothelial growth factor monotherapy for polypoidal choroidal vasculopathy. Br J Ophthalmol. 2018;102(1):97-101.
